# Cystic Urachus

**Published:** 1896-06

**Authors:** Jane W. Carroll

**Affiliations:** Buffalo, N.Y.; 285 Ashland Avenue


### CYSTIC URACHUS.

By JANE W. CARROLL, M. D., Buffalo, N.Y.

THE patient was first seen March 23, 1895. She gave the following history :

Mrs. B., aged 34, married nine years ; no conceptions. Had always had good health and was fleshy until twenty-three years of age, when she began to fail in general health and lose flesh. Had an attack of sharp pain in the bowels, followed by diarrhea. Similar seizures continued off and on for five years and were diagnosticated as consumption of the bowels, sometimes as malarial fever and later as gastric fever. During one attack there was inflammation of the bladder, with deposit of urates in the urine.

During one attack, the year previous, there had been bladder trouble, frequent micturition, with small amount of urine containing uric acid crystals. There was severe abdominal pain this time and the diagnosis was peritonitis.

On March 23, 1895, the patient's condition was a weak one. She was very slender, weighing about a hundred pounds, emaciated, sallow, almost cachectic. Complained of pain in right iliac region, extending from spine of ileum down to pubes, with great tenderness on palpation. There was also a feeling of soreness in the bladder, frequent micturition, and only a small quantity of urine. The patient was afraid to eat solids, as she thought they increased the pain in the bowels, and had cut down all food to a limited amount of bread and butter.

*Examination.*—On external palpation of the bowels found great tenderness in the right iliac region, from the anterior superior spine of the ileum to the pubes. Found in the umbilical region an apparent induration or thickening of the abdominal wall, which gave the impression of some abnormal condition. This induration was found extending from the umbilicus for two or three inches to the right and down three or four inches, forming a parallelogram, lying to the right of the median line.

Per vaginam, was found a contracted pelvis, the examining finger touching the promontory of the sacrum very easily. The uterus was anteverted and held firmly in that position by the contracted pelvis, so that the os could not be brought into view by the speculum. The cervix was congested and slightly enlarged and the parts were feverish to the touch. A bimanual examination excluded any connection between the tumor and the uterus. The explanation of the pain and the tumor then seemed to be confined to an appendicitis, floating kidney, or the pain alone to renal colic. As the tumor maintained its position under different attitudes and on different occasions, floating kidney was excluded, although the shape of the tumor was very much like that of a kidney. McBurney's tender point was not noted. The pain was more widespread and the tumor seemed too near the umbilicus to be of appendicitic origin. The thought of a patulous urachus had presented itself, but as bimanual palpation, per vaginam, between bladder and tumor, did not elicit any pain or distress the idea was abandoned. The first examination of the urine for twenty-four hours showed the amount small, only about a pint ; a later specimen contained urates. Unfortunately the record has been mislaid or more exact data could be given. While waiting to determine, if possible, the nature of the tumor, attention was directed toward the patient's general health. The kidneys were stimulated, tonics and antilithics prescribed. Also a nutritious, easily digested diet, with the result of great improvement for one week or so. Then a return of the old symptoms, great pain in the right iliac region with temperature 101°. This attack lasted five days, with a return to health for another week. April 11th ushered in a recurrence with temperature 101° ; April 12th, temperature 102° ; April 13th, temperature 103°. During this time the patient's mind had been familiarised with the idea that at some time surgical interference would be necessary. The daily increase of temperature, together with a perceptible bulging of the tumor above the contour of the abdomen, also a gurgling on pressure, seemed to indicate that that time had come. Accepting the previous history of inflammation, the theory was plausible that adhesions had been formed between the intestines, omentum and abdominal wall, which caused pain during peristalsis.

The daily rise in temperature now suggested abscess and I requested to be allowed to call a surgeon for consultation. Accordingly, Dr. Roswell Park saw the patient with me on April 14th. As the temperature had now fallen to 100.5°, abscess was excluded. No diagnosis was made, but the patient was advised to go to the hospital for an exploratory operation at least.

This attack subsided in a few days and, after a week of tonic treatment, Mrs. B. entered the general hospital, April 24th, with premonitory symptoms of another attack. April 25th, chloroform having been given, a small incision was made in the median line below the umbilicus, the tissue being very dense and difficult to cut. A sac was cut into, from which some fluid escaped. The incision was enlarged and the examining finger passed into the abdomen. It was found then that the tumor was a cystic urachus ; the connection with the bladder could be traced, but a probe could not be passed. This connection was tied off and the cyst dissected out. A number of adhesions had formed between the tumor, omentum and walls of the abdomen and there was considerable soft new tissue formation, rather gelatinous in character. The peritoneum was adherent to the abdominal wall and a considerable piece of both was removed either side of the incision ; the abdominal walls could, however, be easily approximated and the wound closed.

The patient made an uninterrupted recovery and left the hospital in two weeks. She has had excellent health ever since, and when last seen—April 9, 1896,—was rosy-cheeked and the picture of health.

The probable explanation of the attacks, therefore, seems to be an oozing of urine into the upper or cystic part of the urachus, and as there was no egress for the fluid once gathered, it was absorbed into the system, causing a toxemia, which would account for the temperature. The pain was caused by the dragging, due to the distention. Inflammation must have been present some time, and the adhesions and new tissue formation limited the intestinal action to some extent, and gave rise to much the same symptoms that constricting bands will often cause.

285 Ashland Avenue.

